# Filter-Aided Sample Preparation Procedure for Mass Spectrometric Analysis of Plant Histones

**DOI:** 10.3389/fpls.2018.01373

**Published:** 2018-09-19

**Authors:** Dominika Ledvinová, Kamil Mikulášek, Hana Kuchaříková, Sylva Brabencová, Miloslava Fojtová, Zbyněk Zdráhal, Gabriela Lochmanová

**Affiliations:** ^1^Mendel Centre for Plant Genomics and Proteomics, Central European Institute of Technology, Masaryk University, Brno, Czechia; ^2^National Centre for Biomolecular Research, Faculty of Science, Masaryk University, Brno, Czechia

**Keywords:** histone derivatization, filter-aided sample preparation, post-translational modifications, epigenetics, mass spectrometry, *Arabidopsis thaliana*

## Abstract

Characterization of histone post-translational modifications (PTMs) is still challenging, and robust histone sample preparation is essential for convincing evaluation of PTMs by mass spectrometry. An effective protocol for extracting plant histone proteins must also avoid excessive co-extraction of the numerous potential interfering compounds, including those related to secondary metabolism. Currently, the co-existence of histone marks is addressed mostly by shotgun proteomic analysis following chemical derivatization of histone lysine residues. Here, we report a straightforward approach for plant histone sample preparation for mass spectrometry, based on filter-aided sample preparation coupled with histone propionylation. The approach offers savings in sample handling and preparation time, enables removal of interfering compounds from the sample, and does not require either precipitation or dialysis of histone extract. We show the comparison of two protocol variants for derivatization of histone proteins, in-solution propionylation in the vial and propionylation on the filter unit. For both protocols, we obtained identical abundances of post-translationally modified histone peptides. Although shorter time is required for histone protein labeling on the filter unit, in-solution derivatization slightly outweighed filter-based variant by lower data variability. Nevertheless, both protocol variants appear to be efficient and convenient approach for preparation of plant histones for mass spectrometric analysis.

## Introduction

Plants have evolved complex, highly efficient, and flexible epigenetic regulation mechanisms, including a broad suite shared with other eukaryotes. In addition, they have acquired various specialized traits reflecting the sessile nature of their lifestyle (Pikaard and Mittelsten Scheid, 2014). They exhibit considerable morphological and physiological plasticity in response to stimuli from the surrounding environment, due to rapid transcriptional changes mediated by epigenetic chemical modifications, such as DNA methylation and histone post-translational modifications (hPTMs), and by the regulation activity of small and non-coding RNA molecules. In addition, epigenetic modifications persist through cell divisions, providing a molecular memory that underpins the maintenance phase of these responses (Baulcombe and Dean, 2014).

Complexity of histone variants together with PTM combinations represent a challenge in studying epigenetic regulation (reviewed by El Kennani et al., 2018). To investigate histone modification states, various strategies based on immunological procedures or mass spectrometry (MS) are available. While ChIP-based methods (e.g., ChIP-seq or ChIP-on-chip) are favored for analysis of histone marks at functionally distinct chromatin (Ha et al., 2011; Roudier et al., 2011; Sequeira-Mendes et al., 2014; Zhang et al., 2015), MS-based analysis is better suited to study of a system-level view of histone modifications (Johnson et al., 2004; Chen et al., 2015; Moraes et al., 2015). In addition to the identification and quantification of histone marks, unlike specific antibodies MS enables detection of novel hPTMs (Strahl et al., 2001; Gardner et al., 2011). Moreover, the combinatorial nature of hPTMs can be explored in intact histones using a top-down MS strategy or in large peptides using a middle-down approach (Moradian et al., 2014). In general, a bottom-up MS is more frequently used approach for protein identification and quantification as it offers greater sensitivity and mass accuracy, and less demanding data analysis. Histones, enriched in lysine and arginine residues, present a challenge for bottom-up approach due to the generation of numerous small hydrophilic peptides after trypsin digestion, with poor chromatographic retention and unambiguous localization of the present PTMs. To circumvent this issue, a recent protocol was proposed, which includes lysine chemical derivatization. Such derivatization blocks the ε-amino groups of unmodified and monomethyl lysine residues, allowing trypsin to perform proteolysis only at the C-terminal of arginine residues (Garcia et al., 2007; Plazas-Mayorca et al., 2009). Post-digestion labeling of newly generated N-termini of histone peptides improves chromatographic retention and detection during liquid chromatography-tandem MS (LC-MS/MS). The most popular chemical derivatization technique used for hPTM characterization is propionylation, although certain technical drawbacks were disclosed, i.e., incomplete or non-specific derivatization at serine, threonine, and tyrosine (Meert et al., 2015, 2016). The utility of derivatizing agents with various chemical structures, such as propionic anhydride and propionic acid *N*-hydroxysuccinimide ester (NHS-propionate), has been extensively evaluated under diverse conditions to optimize the procedure (Lin and Garcia, 2012; Liao et al., 2013; Meert et al., 2015). Improvements in retention of hydrophilic peptides, e.g., H3K4me2/me3, and recoveries have been achieved by hybrid histone labeling using propionic anhydride for protein derivatization followed by post-digestion labeling of peptide N-termini using phenylisocyanate (PIC; Maile et al., 2015). In-gel variants of propionylation have also been developed, based on either a standard protocol using propionic anhydride or NHS-propionate reagent (Ouvry-Patat and Schey, 2007; Chen et al., 2015).

As plants and mammals have acquired similar mechanisms of epigenetic regulation, standard MS-based approaches for hPTM characterization are broadly applicable for all taxa, except preparation of histone extracts. Extracting histone proteins from plant tissues is challenging due to the presence of diverse potentially contaminating compounds. Frequently used protocols for extracting histones from mammalian cells include cell disruption and solubilization using detergent and a chaotropic agent, followed by extraction of histone proteins into an acidic solution and finally precipitating them. Although not yet reported directly, most plant histone precipitates are difficult to re-dissolve (substantially more difficult to dissolve than mammalian histones). Indeed, issue of histone solubility differs among plant species as evident from previously published extraction protocols; e.g., sonication is sufficient to dissolve histones from cauliflower in water while strong detergents and chaotropic agents are commonly added to dissolve histone pellets from Arabidopsis (Jackson et al., 2004; Mahrez et al., 2016). Such chemical additives are incompatible with downstream MS analysis and must be removed. Alternatively, the precipitation step is omitted and time-consuming dialysis of histone extract against acidified water is performed (Waterborg et al., 1987; Bergmüller et al., 2007).

Difficulties encountered during plant histone sample preparation for MS are reflected by the limited number of studies focused on plant histone characterization. Moreover, only a few studies dealing with plant hPTM used the advantage of shotgun proteomics coupled with lysine derivatization which is widely used for mammalian histone analyses (Johnson et al., 2004; Chen et al., 2015; Moraes et al., 2015). Johnson’s and Chen’s groups performed prefractionation of histone samples isolated from Arabidopsis leaves prior propionylation of histone H3. While Johnson et al. (2004) purified histone H3 N-termini from dialyzed crude histone extract using double-round HPLC, Chen et al. (2015) separated histone sample after acidic extraction using SDS–PAGE and performed NHS-propionate derivatization in gel pieces corresponding to histone H3. Moraes et al. (2015) omitted histone fractionation and used dialyzed total histone crude extract from sugarcane for propionylation to characterize PTMs at histone H3 and H4 by LC-MS/MS.

As evident, neither prevailing nor straightforward protocol for plant histone preparation for mass spectrometric analysis including histone derivatization has been published so far. In the present study, we established a protocol for preparing samples of plant histones prior to LC-MS/MS analysis, using a filter-aided sample preparation (FASP) technique to remove natural contaminants and chemical additives incompatible with MS-based procedures (Wiśniewski et al., 2009). We included the derivatization protocol into histone sample preparation workflow described in our previous study (Brabencová et al., 2017). The final workflow consists of nuclei isolation, histone extraction into acidic solvent, protein propionylation and on-membrane digestion, then peptide propionylation in solution. Here, we especially aimed at plant histone derivatization as a specific step of sample preparation for mass spectrometric analysis. Two variants of the derivatization protocol, propionylation of histone proteins either in solution (PROP-in-SOL) or on a filter unit (PROP-on-FILTER), were evaluated according to their derivatization efficiency, time demands, and data variability. Both alternatives, PROP-in-SOL or PROP-on-FILTER, raise the possibilities of histone characterization in plant epigenetic studies. Importantly, PROP-in-SOL variant was proved to be suitable also for hPTM characterization in young Arabidopsis seedlings.

## Materials and Methods

### Plant Material and Growth Conditions

*Arabidopsis thaliana* ecotype Columbia 0 seeds were sterilized by ethanol, sown on half-strength Murashige–Skoog medium (Duchefa Biochemicals) agar plates, then germinated in phytotrons under short day conditions (8 h light, with 100 mmol m^-2^ s^-1^ illumination at 21°C and 16 h dark at 19°C). After 7 days, seedlings were transferred to soil and plants were grown for 6 weeks under short day conditions.

### Histone Extraction From Plant Material

*Arabidopsis thaliana* 7 weeks old leaves (∼500 mg) or seven days old seedlings (∼300–500 mg) were ground in liquid nitrogen and homogenized in extraction buffer I (10 mM NaCl, 10 mM 2-(N-morpholino)ethanesulfonate pH 6.0, 5 mM EDTA, 0.25 M sucrose, 0.6% Triton X-100, 0.2 M spermidine, 100 mM PMSF, 45 mM sodium butyrate and 20 mM β-mercaptoethanol) to “soft ice” consistency. The homogenate was filtered through nylon mesh and centrifuged (10 min, 3000 g, 4°C). The pellet was washed twice with the extraction buffer, resuspended in Percoll buffer (2.4 g of 5× concentrated extraction buffer, 18 g of Percoll from Sigma-Aldrich) and centrifuged (15 min, 4000 g, 4°C). Nuclei floating on the Percoll buffer surface were collected, washed three times by resuspension in washing buffer (75 mM NaCl, 10 mM EDTA, 50 mM Tris–HCl, pH 8.0) and centrifugation (10 min, 3000 g, 4°C). Nuclei were then resuspended in extraction buffer II (50 mM Tris–HCl pH 8.0, 100 mM NaCl, 3 mM EDTA, 1% CHAPS, 0.1 mM PMSF, 45 mM sodium butyrate, and 10 μl mL^-1^ P9599 protease inhibitor cocktail from Sigma-Aldrich), incubated for 1 h on ice, and centrifuged (8 min, 10 000 g, 4°C). The pellet was resuspended in 200–400 μL of ice-cold 0.2 M H_2_SO_4_ and incubated overnight, with shaking at 4°C. The sample was then centrifuged (8 min, 16100 g, 4°C) and the supernatant containing histone proteins was collected. Protein concentration was measured in aliquots sixteen times diluted with deionized water, using a Micro BCA^TM^ Protein Assay Kit.

### Chemical Derivatization of Histone Proteins

#### Protocol Variant I: In-Solution Protein Propionylation (PROP-in-SOL)

Histone samples were subjected to a double round of propionic anhydride derivatization, as previously described (Sidoli et al., 2016), with modifications. Briefly, a 16 μg portion of plant histone extract in sulfuric acid was taken into the vial and pH was adjusted to 8–9 by NH_4_OH. A 10 μL portion of propionylation reagent, freshly prepared for each batch of three samples by mixing propionic anhydride and acetonitrile (MeCN) in a 1:3 ratio, was immediately added to the sample. The pH was adjusted to 8–9 by NH_4_OH, then the sample was incubated in a thermomixer (37°C, 700 rpm, 20 min) and the sample volume was reduced in a Savant SPD121P concentrator (SpeedVac; Thermo Scientific) to 5 μL. For the second round of propionylation, the sample was diluted with 50% (v/v) MeCN to a volume of 20 μL. The second round of propionylation was carried out with the same protocol. FASP was used for protein digestion to remove both natural and chemical contaminants. The sample was diluted with 300 μL of 8 M urea (pH 8.5), placed in a YM-10 Microcon filter unit (Millipore), centrifuged (14000 g, 45 min, 25°C) and washed two times with 200 μL of 8 M urea.

#### Protocol Variant II: On-Membrane Protein Propionylation (PROP-on-FILTER)

A 16 μg of plant histone extract in sulfuric acid was placed in a YM-10 Microcon filter unit (Millipore) with 200 μL of 100 mM ammonium bicarbonate (ABC; pH 8.0), centrifuged (14000 g, 20 min, 25°C) and washed three times with 200 μL of 100 mM ABC. The sample was diluted with 100 mM ABC to a volume of 30 μL then 2 μL of NH_4_OH was added. A 20 μL portion of propionylation reagent, freshly prepared for each batch of three samples by mixing propionic anhydride and isopropanol in a 1:3 ratio, was immediately added to the sample. The pH was adjusted to 8–9 by NH_4_OH, then the sample was incubated in a thermomixer (37°C, 700 rpm, 20 min) and centrifuged (14000 g, 15 min, 25°C). The second round of propionylation was carried out with the same protocol.

### On-Membrane Proteolytic Digestion and In-Solution Peptide Propionylation

The sample on the membrane was washed three times with 100 μL of 100 mM ABC (14000 g, 45 min, 25°C), and trypsin diluted in 50 μL of 100 mM ABC was added in a 1:40 enzyme:protein ratio. Following overnight digestion at 37°C, the digest was collected by centrifugation (14000 g, 10 min, 25°C), subjected to two additional washes with 50 μL of 100 mM ABC, then concentrated in the SpeedVac to ∼20 μL.

One microliter of NH_4_OH was added to the sample, then 5 μL of the propionylation reagent prepared by mixing propionic anhydride with MeCN in a 1:3 ratio was added. The pH was adjusted to 8–9 by NH_4_OH, the sample was incubated in thermomixer at 37°C at 700 rpm for 20 min, then the sample volume was reduced in the SpeedVac to 5 μL. For the second round of propionylation, the sample was diluted with 50% (v/v) MeCN to a volume of 20 μL. The second round of propionylation was carried out with the same protocol. The sample was diluted with 0.1% formic acid (FA) to a volume of 100 μL. Labeled histones were desalted using a HyperSep SpinTip C-18 column (Thermo Fisher Scientific), and the peptide concentration was determined using a Micro BCA^TM^ Protein Assay Kit.

### Mass Spectrometric Analysis, Database Searches and Quantification of Histone Peptide Forms

Propionylated peptides were measured using liquid chromatography-tandem mass spectrometry (LC-MS/MS). The LC-MS/MS equipment consisted of an RSLCnano system, equipped with an X-Bridge BEH 130 C18 trap column (3.5 μm particles, 100 μm × 30 mm; Waters), and an Acclaim PepMap100 C18 analytical column (3 μm particles, 75 μm × 500 mm; Thermo Fisher Scientific), coupled to an Orbitrap Elite hybrid spectrometer (Thermo Fisher Scientific) equipped with a Digital PicoView 550 ion source (New Objective) using PicoTip SilicaTip emitter (FS360-20-15-N-20-C12), and Active Background Ion Reduction Device. Prior to LC separation, tryptic digests were online concentrated on trap column. The mobile phase consisted of 0.1% formic acid in water (A) and 0.1% formic acid in 80% acetonitrile (B), with the following proportions of B: 1% for 3 min at 500 nl/min, then with a switch to 300 nl/min for next 2 min, increased linearly from 1 to 70% over 85 min, 70–85% over 20 min and followed by isocratic washing at 85% B for 10 min. Equilibration with 99:1 (mobile phase A:B; flow rate 500 nl/min) of the trapping column and the column was done prior to sample injection to sample loop. The analytical column outlet was directly connected to the ion source of the MS. MS data were acquired using a data-dependent strategy selecting up to top 10 precursors based on precursor abundance in a survey scan (350–2000 m/z). The resolution of the survey scan was 60000 (400 m/z) with a target value of 1 × 10^6^, one microscan and maximum injection time of 1000 ms. HCD MS/MS spectra were acquired with a target value of 50000 and resolution of 15000 (400 m/z). The maximum injection time for MS/MS was 500 ms. Dynamic exclusion was enabled for 45 s after one MS/MS spectrum acquisition and early expiration was disabled. The isolation window for MS/MS fragmentation was set to 2 m/z.

The RAW mass spectrometric data files were analyzed using Proteome Discoverer software (Thermo Fisher Scientific; version 1.4) with in-house Mascot search engine (Matrix Science, version 2.6) to compare acquired spectra with entries in the UniProtKB *A. thaliana* protein database (version 2017_11; 27567 protein sequences; downloaded from^1^), cRAP contaminant database (downloaded from^2^) and in-house histone database (version 2017_02; 71 protein sequences). Mass tolerances for peptides and MS/MS fragments were 7 ppm and 0.025 Da, respectively. Semi-Arg-C for enzyme specificity allowing up to two missed cleavages was set. For searches against cRAP and UniProtKB *A. thaliana* databases the variable modification settings were oxidation (M), deamidation (N, Q), acetylation (K, protein N-term) and propionylation (K, N-term), while for histone database searches they were acetylation (K, protein N-term), methylation (K, R), dimethylation (K), trimethylation (K), phosphorylation (S, T), and propionylation (K, N-term, S, T, Y). The abundance of histone peptides was quantified automatically using Proteome Discoverer 1.4 software. Only peptides with statistically significant peptide score (*p* < 0.01) were considered. The peak area corresponding to each precursor ion was calculated from the extracted ion chromatograms (XICs) using the Precursor Ions Area Detector node. Selected histone peptide identifications were manually verified and quantified from the peak areas derived from the XICs using Skyline 3.6 software, including identification alignment across the raw files based on retention time and m/z. A representative separation of positional isomers is illustrated in **Supplementary Figure 1**. Those positional isomers which were not baseline separated were quantified as mixture (e.g., K18ac/K23ac). The relative abundance (RA) of individual histone H3 and H4 post-translationally modified peptides was calculated by dividing the XIC peak areas for the corresponding assignable products by the sum of the peak areas representing the total pool of all quantified H3 or H4 peptides:

$$RA=\frac{\sum \mathrm{peak}\;\mathrm{areas}\;\mathrm{of}\;\mathrm{XIC}\;\mathrm{for}\;\mathrm{peptides}\;\mathrm{with}\;\mathrm{certain}\;\mathrm{PTM}\;\mathrm{pattern}}{\sum \mathrm{peak}\;\mathrm{areas}\;\mathrm{of}\;\mathrm{XIC}\;\mathrm{of}\;\mathrm{all}\;\mathrm{quantified}\;\mathrm{peptides}}$$

The mass spectrometry proteomics data have been deposited to the ProteomeXchange Consortium via the PRIDE (Vizcaíno et al., 2016) partner repository with the dataset identifier PXD010102.

## Results

**FIGURE 1:**
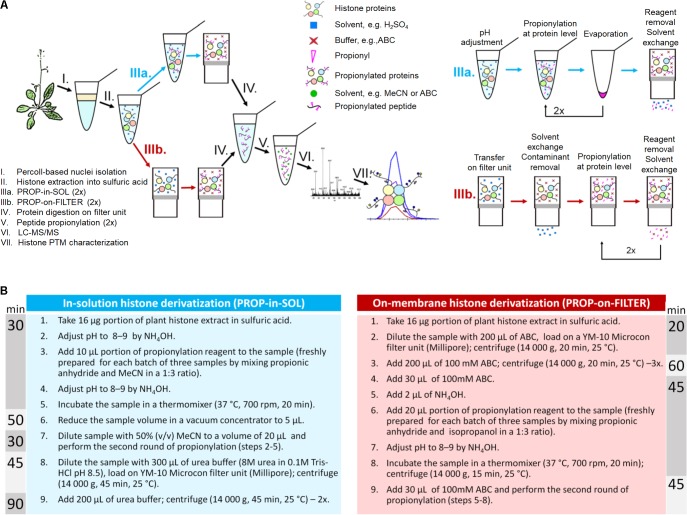
Plant histone sample preparation workflow for LC-MS/MS. **(A)** An illustrative scheme of the workflow. **(B)** Protocol variants for protein derivatization; time needed for each derivatization protocol is depicted.

Given the nature of plant samples, an appropriate protocol for histone preparation for “bottom-up” proteomic analysis was designed. The performance of histone derivatization procedure in complex plant samples was evaluated. The derivatization protocol was included into histone sample preparation workflow described in the previous study of Brabencová et al. (2017) (the complete workflow is described and schematically illustrated in **Figure 1**). Unlike smooth histone preparation from mammalian cell cultures, histone extraction from plant tissues for LC-MS/MS is more challenging due to the presence of plant species-specific contaminants such as proteins, polysaccharides, polyphenols, and secondary metabolites. Compared to high-purity nuclei isolated from mammalian cells, plant isolates usually contain nuclei along with a considerable amount of starch granules. Besides, in case of a low amount of starting material (e.g., a young seedling), histone yield is further determined by limited nuclei amount. Moreover, quantitative solubilization of histone precipitate is hard to accomplish in certain plant species. The presented plant histone preparation workflow for LC-MS/MS deals with above-described difficulties (plant-specific features of histone preparation is illustrated in **Supplementary Figure 2**).

### FASP-Based Preparation of Histone Proteins for Shotgun Proteomic Analysis

A chemical derivatization technique of proven value for histone analysis was adapted for derivatization of plant acidic histone extracts. Initially, PROP-on-FILTER protocol was thoroughly tested using histones extracted from mammalian cells. This material was chosen because of the simplicity of the sample preparation process (straightforward histone extraction and subsequent trouble-free solubilization of histone proteins in water). The method performance was assessed by comparison with a commonly used in-solution derivatization approach according to Sidoli et al. (2016); see **Supplementary Data** for details. Comparable performance of two methods was observed by strongly overlapping profiles of majority of identified histone peptide forms and their RAs as well as by the efficiency of the labeling (**Supplementary Figure 3**). Such results provided opportunities for easy introducing of PROP-on-FILTER to plant histone preparation procedure to enhance the uniformity and hydrophobicity of acquired plant histone peptides, and simplify the procedure by performing several sample preparation steps in one reactor (i.e., buffer exchange, chemical derivatization, and protein digestion).

PROP-on-FILTER method was compared with an in-solution derivatization, PROP-in-SOL, by evaluation of hPTMs abundance in *A. thaliana* samples. Histone extracts obtained from five biological replicates were processed using each protocol. In PROP-in-SOL method, pH of acidic histone extract was adjusted to 8 and propionylation of histone proteins occurred in a vial. The PROP-on-FILTER method used the membrane to exchange sulfuric acid for ABC buffer and protein derivatization was performed directly on the membrane. Both approaches used the membrane for reagent removal, buffer exchange and protein digestion. Filter units with 10 kDa cut-off were used as they had sufficiently small membrane pores to retain histone proteins (MW ∼11–29 kDa), while allowing residual unreacted derivatization reagent to pass through. Due to limited membrane chemical compatibility, ABC was favored over MeCN for washing samples, as well as for use as a reaction buffer for propionylation labeling in PROP-on-FILTER method. Two times higher proportion of the propionylation reagent prepared by mixing propionic anhydride with isopropanol was added, relative to the amount of total protein, than in the PROP-in-SOL procedure to ensure high efficiency of the reaction in the filter unit. On the other hand, sample wash with urea buffer was omitted in PROP-on-FILTER method to reduce the number of steps and also less time was needed for reagent removal by ultrafiltration compared to evaporation in PROP-in-SOL procedure. The overall time required to execute PROP-in-SOL and PROP-on-FILTER protocol was 245 min and 170 min, respectively.

### Efficiency of Histone Proteins/Peptides Labeling

**FIGURE 2:**
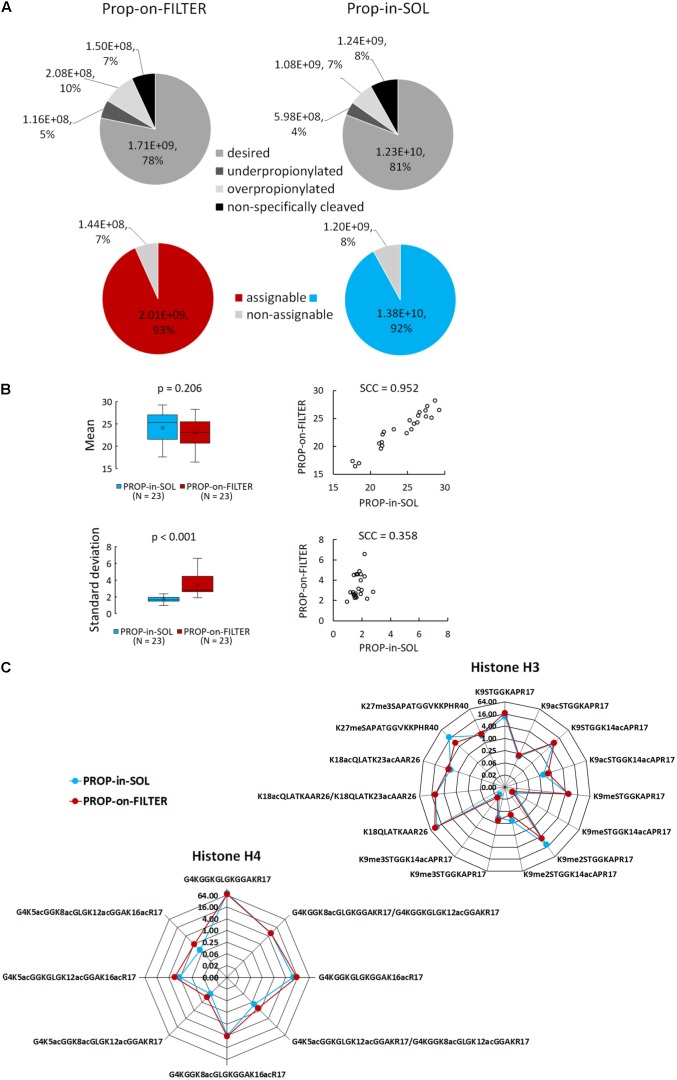
Comparison of PROP-in-SOL and PROP-on-FILTER performance. **(A)** Grayscale pie charts showing proportions of identified histone H3 and H4 peptides in four categories – desired (peptides cleaved and propionylated as expected), underpropionylated (peptides with at least one unmodified amino group on lysine residue or N-terminus), overpropionylated (peptides with at least one propionylated hydroxyl group on S, T, or Y residue), non-specifically cleaved (peptides with cleavage at lysine C terminus or missed cleavage at arginine C terminus). Color pie charts showing proportions of assignable peptides, i.e., peptides enabling correct quantification. **(B)** Box-plots and scatter-plots of the means and standard deviations of abundances of histone H3 and H4 peptide forms detected in the samples. The box-plots show extremes, interquartile ranges and medians (*N* = 23). Means and standard deviations were compared by Mann-Whitney tests (*p*-values) and Spearman’s correlation coefficients (SCC values). **(C)** Radar charts showing relative abundances of individual peptide forms of histone H3 and H4 (medians; *N* = 5), determined from the ratio of the XIC peak areas of particular assignable products to the summed XIC peak areas of the total pool of all quantified H3 or H4 peptides, respectively. The Y axes have a binary logarithm scale, with zero located in the center.

PROP-in-SOL and PROP-on-FILTER protocols’ performance in terms of propionylation efficiency was compared. Similar numbers of histone peptide forms were detected by LC-MS/MS following both procedures. Identified peptides of H3 and H4 histones were manually tagged in extracted ion chromatograms (XIC) in Skyline software and quantified. The peptides were sorted into four categories: (1) desired – peptides cleaved and propionylated as expected; (2) underpropionylated – missed propionylation on lysine residue or N-terminus; (3) overpropionylated – propionylation on hydroxyl group of serine (S), threonine (T) or tyrosine (Y); and (4) non-specifically cleaved – amino acid sequence cleaved at lysine C-terminus due to insufficient propionylation on protein level or missed cleavage at arginine C-terminus. The precursor peak areas of the peptides in each category were summed, and the percentages of desired, underpropionylated, overpropionylated and non-specifically cleaved peptides were found to be 78.0, 5.0, 10.0, and 7.0%, respectively, in PROP-on-FILTER samples, and 81.0, 4.0, 7.0, and 8.0%, respectively, in PROP-in-SOL samples (**Figure 2A**). Thus, the percentage of desired peptides in the PROP-on-FILTER samples was comparable with those in the PROP-in-SOL samples. As a considerable percentage of unintended peptides (in categories 2–4) can be used for quantification of corresponding histone peptide forms (hereafter referred to as “assignable”), the final proportion of assignable peptides obtained by both procedures was >90%, while the remaining peptides were unassignable peptides cleaved at the lysine C-terminus (**Figure 2A**).

### Abundance of Histone Peptide Forms

For each preparation method, inter-sample variability in abundance of histone peptide forms was assessed. In total, 15 and 8 peptide forms from N-termini of histones H3 and H4 (aa sequence regions 9–40 and 4–17, respectively) identified using both methods were selected for evaluation (**Supplementary Table 1**). The difference in mean log_2_-transformed peptide precursor areas obtained from analyses of samples prepared by the two protocols was not significant (*p* = 0.206), but standard deviations of PROP-on-FILTER samples were significantly higher than those of PROP-in-SOL samples (*p* < 0.001; **Figure 2B**). Moreover, mean precursor areas were highly correlated, but not their standard deviations (Spearman correlation coefficients, 0.952 and 0.358, respectively). Noticeably, higher inter-sample variability related to PROP-on-FILTER method was observed, especially in case of H4 peptide forms (**Supplementary Figure 4**). The comparison was then extended to relative quantification of the same histone peptide set. The RAs of most peptides in samples derivatized by the two methods were very similar, but there were a few exceptions (**Figure 2C**). There were two peptides with markedly lower abundance in the samples prepared by the PROP-on-FILTER procedure: KSTGGKAPR (H3, aa 9–17) in modification state K9me2 and KSAPATGGVKKPHR (H3, aa 27–40) in modification state K27me1. However, there was also one peptide with markedly lower abundance in samples prepared by the PROP-in-SOL procedure: GKGGKGLGKGGAKR (H4, aa 4–17) in tetra-acetylated state. Nevertheless, the strongly overlapping profiles of majority of identified histone peptide forms and their RAs show that the two methods have comparable performance.

### Characterization of Histone PTM in Young Seedlings

**FIGURE 3:**
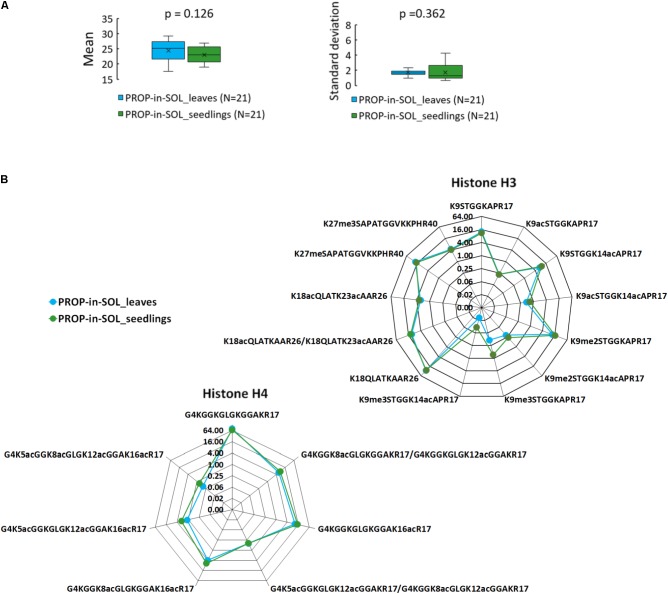
PROP-in-SOL performance for hPTM characterization in different plant developmental stages. **(A)** Box-plots of the means and standard deviations of abundances of histone H3 and H4 peptide forms detected in the seedling and leaf samples. The box-plots show extremes, interquartile ranges and medians (*N* = 21). Means and standard deviations were compared by Mann-Whitney tests (*p*-values). **(B)** Radar charts showing relative abundances of individual peptide forms of histone H3 and H4 (medians; *N* = 5), determined from the ratio of the XIC peak areas of particular assignable products to the summed XIC peak areas of the total pool of all quantified H3 or H4 peptides, respectively. The Y axes have a binary logarithm scale, with zero located in the center.

To verify the suitability of the method for hPTM characterization in young seedlings, one of the most critical stage of the plant development, 7-day-old Arabidopsis seedlings were processed using the proposed sample preparation workflow. Considering the lower variability of the data, PROP-in-SOL was favored over PROP-on-FILTER approach for histone derivatization. Inter-sample variability in abundance of histone peptide forms was assessed using 14 and 7 peptide forms from N-termini of histones H3 and H4 (aa sequence regions 9–40 and 4–17, respectively; **Supplementary Table 2**). The mean log_2_-transformed peptide precursor areas of seedling samples were slightly lower compared to leaf samples, however, the difference was not significant (*p* = 0.126; **Figure 3A** and **Supplementary Figure 5**). Similarly, the standard deviations between seedling and leaf samples were comparable (**Figure 3A**; *p* = 0.362). These data together with similar RAs profile of observed peptides (**Figure 3B**) demonstrate that presented method of histone sample preparation is usable on different developmental stages of plants.

## Discussion

Plants natural ability to adapt rapidly to myriads of changes in environmental conditions is conferred by intricate webs of metabolic pathways and large numbers of associated primary and secondary metabolites. Thus, in plant extracts, there are numerous substances potentially interfering with MS analysis, and more purifying steps must be included in protocols used to extract histones from plant tissues than in protocols used for simpler matrices. For example, after cell wall disruption and histone extraction from lysed nuclei, histones can be prepared by precipitation, or dialysis against acidified water (Jackson et al., 2004; Bergmüller et al., 2007; Mahrez et al., 2016). Another widely used approach, which avoids the nuclei isolation step, involves purification of histones using the weak cation exchanger Bio-Rex-70 and dialysis (Waterborg et al., 1987). Recently reported multistep extraction protocol for plant linker histones further demonstrates the obstacles inherent in green plants (Kotliński et al., 2016).

Currently, derivatization of amine groups in both the side-chain of lysines and the peptide N-termini is widely employed to further prepare histone samples for LC-MS/MS analysis. Effects of various factors in propionylation of mammalian histones have been previously evaluated, e.g., solvents, buffers, incubation time and temperature, and numbers of derivatization rounds. Several technical drawbacks of the procedure have been reported, including incomplete or non-specific derivatization, insufficient increases of hydrophobicity by the labeling, and bias in ionization efficiency (Liao et al., 2013; Meert et al., 2015, 2016; Sidoli et al., 2015). Various other acid anhydrides (including candidates with ring structures that eliminate uncontrolled chemical side reactions caused by drops in pH) have been used in attempts to avoid these potential pitfalls (Sidoli et al., 2015). However, peptide propionylation still appears to be the most efficient available derivatization procedure, in terms of hydrophobicity gained, ionization efficiency and signal intensity. In several studies, histone labeling using deuterated acetic anhydride was favored over propionylation to obtain peptides with similar physicochemical properties to those bearing endogenous acetylation (Smith, 2005; Nakayasu et al., 2014; Feller et al., 2015). In a recently reported FASIL-MS method, the succinimide ester chemistry of N-acetoxy-D3-succinimide combined with FASP protocol showed a higher efficiency in lysine derivatization at protein level compared to the conventional propionic anhydride method in case of mammalian histone extract (Vitko et al., 2016). Indeed, both acetylation as well as propionylation deal with positional isomers co-elution problem during chromatography (e.g., diacetylated G4 – R17 peptide at histone H4 possessing four lysines offers six possible combinatorial variants with closed physico-chemical properties). In this regard, the implementation of targeted MS1-MS2-MS3 scans represented key achievement in quantification of positional isomers in histone acetylome study in *Drosophila* cells (Feller et al., 2015; Blasi et al., 2016). In our plant histone preparation protocol, we utilized propionic anhydride, the most proven agent for labeling of amine groups in both the side-chain of lysines and the peptide N-termini. The former was incorporated into FASP-based sample preparation. FASP is a proteomic strategy of sample preparation allowing removal of contaminant and enzymatic digestion (Wiśniewski et al., 2009). In addition, it is useful for removing excess of reagent used for chemical derivatization of proteins (Wiśniewski and Pruś, 2015; Vitko et al., 2016). In comparison to mammalian histones of relatively high purity, plant extracts are usually more complex, and their protein composition vary considerably depending on the way of sample preparation used (Brabencová et al., 2017). Indeed, an inappropriate histone:reagent ratio may lead to either a high amount of residual unreacted reagent or insufficient derivatization. FASP-based sample preparation is advantageous in this respect, because it allows the derivatization of histone proteins of undefined purity with excess reagent to ensure sufficiently efficient derivatization and subsequent removal of unreacted propionylation reagent. Two protocol variants for plant histone sample preparation for mass spectrometric analysis presented here have been shown to give the same quantitative information on histone modified peptides. Thus, both appear to represent meaningful alternative approaches for plant histone preparation. The main differences between the protocols are summarized in **Supplementary Table 3**. The PROP-in-SOL is preferred approach for histone preparation due to lower inter-sample variability provided which is a crucial parameter for evaluation of changes in hPTM related to certain biological basics (Brabencová et al., 2017). For example, PROP-in-SOL method is more suitable for histone sample preparation from low amount of input material, i.e., young seedlings. PROP-on-FILTER protocol is more straightforward and less time-consuming. Relative abundance, more robust strategy for hPTMs evaluation compared to absolute values, is recommended when PROP-on-FILTER protocol is used to compensate for higher inter-sample variability. Regardless of the method used, the purity of isolated nuclei is a prerequisite for obtaining reliable results.

Development of the sample preparation procedure presented here was prompted by a need for a more straightforward approach for preparing plant histones for “bottom-up” proteomic analysis. The coupling of FASP and propionylation enables reduction of numbers of preparation steps, which often result in sample loss (e.g., protein clean-up by precipitation or dialysis), and considerably decreases the time required for sample preparation (**Table 1**). It also reduces the abundance of contaminants related to the derivatization reagent, which impairs ionization efficiency of targeted peptides. The protocol’s convenience facilitates investigation of changes in histone epigenetic modifications during plant growth, development and environmental interactions.

**Table 1:** Overview of sample preparation methods used for mass spectrometric analysis of plant histones.

Plant histone preparation method	Input material			Histone extraction		Contaminant removal/Prefractionation				Chemical derivatization	Estimate of total time required (days)
				
	Plant species part/stage	Age stage (days/weeks/months)	Fresh weight (g)	Acidic solution	GuCl buffer	Dialysis	Ultra-filtration	HPLC	PAGE	Lysine/N-termini		
Johnson et al., 2004	Arabidopsis inflorescences	N/F	1.0	×	✓	✓	×	✓	×	Propionic anhydride	>7
Chen et al., 2015	Arabidopsis leaves	4 weeks	N/F	✓	×	×	×	×	✓	NHS-propionate	∼4
Moraes et al., 2015	Sugarcane	6 months	40.0	×	✓	✓	×	×	×	Propionic anhydride	∼5
Method variant I Prop-in-SOL	Arabidopsis leaves	7 weeks	0.5	✓	×	×	✓	×	×	Propionic anhydride	∼3
	seedlings	7 days	0.3–0.5								
Method variant II Prop-on-FILTER	Arabidopsis leaves	7 weeks	0.5	✓	×	×	✓	×	×	Propionic anhydride	∼3


*Comparison of proposed method with previously published methods including plant histone derivatization prior LC-MS/MS, showing input material, histone extraction, the way of contaminant removal, the need of prefractionation, derivatizing agent used, and estimate of total time required from tissue homogenization to histone peptide derivatization. N/F, information not found. GuCl buffer, guanidine hydrochloride in 0.1 M potassium phosphate buffer, pH 6.8.*

## Author Contributions

GL, MF, and ZZ contributed to the trial design. DL provided and performed the sample preparation from human culture cells. SB provided all plant material for further processing. SB and MF performed the sample preparation from plant material. KM and HK performed LC-MS/MS analyses of leaf and seedling samples, respectively. DL and GL contributed to the data processing. The manuscript was written with contributions of all authors. All authors have given approval to the final version of the manuscript.

## Conflict of Interest Statement

The authors declare that the research was conducted in the absence of any commercial or financial relationships that could be construed as a potential conflict of interest.

## Footnotes

1 ftp://ftp.uniprot.org/pub/databases/uniprot/current_release/knowledgebase/reference_proteomes/Eukaryota/UP000006548_3702.fasta.gz

2 http://www.thegpm.org/crap/
