# Supplementary material for: Filter-Aided Sample Preparation Procedure for Mass Spectrometric Analysis of Plant Histones
Source: Front Plant Sci. 2018 Sep 19;9:1373. doi: 10.3389/fpls.2018.01373 (PMC6156276; doi:10.3389/fpls.2018.01373)
Supplement: Supplementary file 1 [file Data_Sheet_1.pdf]

Supplementary Material

Filter-aided sample preparation procedure for mass spectrometric analysis of plant histones

Dominika Ledvinová, Kamil Mikulášek, Hana Kuchaříková, Sylva Brabencová, Miloslava Fojtová, Zbyněk Zdráhal, and Gabriela Lochmanová*

*** Correspondence:**
Gabriela Lochmanová
gabriela.lochmanova@ceitec.muni.cz

1 Supplementary Data

1.1 Chemical derivatization technique for histone proteins adapted to on-membrane performance using mammalian histone extracts

1.1.1 In-solution protein propionylation of mammalian histones (PROP-in-SOL_mh) and proteolytic digestion followed by in-solution propionylation of peptides

Histones extracted from MEC-1 cells were subjected to a double round of propionic anhydride derivatization (at both protein and peptide levels). Briefly, a 20 µg portion of histone sample was diluted with acetonitrile (MeCN) and deionized water to a final volume of 20 µL and final MeCN concentration of 50% (v/v). NH_4OH (1 µL) was added, then propionylation reagent was prepared by mixing propionic anhydride with MeCN in a 1:3 ratio and a portion equal to 25% of the sample volume was immediately added. The pH was adjusted to 8–9 by NH_4OH, the sample was incubated in thermomixer at 25 °C and 750 rpm for 20 min, then the sample volume was reduced in a Savant SPD121P concentrator (SpeedVac; Thermo Scientific) to 5 µL. The second round of propionylation was carried out with the same protocol. Propionylated histone proteins were reconstituted in 50 µL of 50 mM triethylammonium bicarbonate (TEAB) and trypsin was added in a 1:40 enzyme:protein ratio. After overnight digestion at 37 °C, the sample was dried in the SpeedVac. The generated peptides were subjected to a double round of propionylation at N-termini using the protocol described above. Each sample was dried in a SpeedVac overnight and dissolved in 50 µL of 50% MeCN. The resulting solution was concentrated in the SpeedVac to 20 µL and its peptide concentration was determined using a Micro BCA™ Protein Assay Kit. Prior to LC-MS/MS analysis, the sample was acidified by adding formic acid (FA) to a final concentration of 1%.

1.1.2 On-membrane protein propionylation of mammalian histones (PROP-on-FILTER_mh) and proteolytic digestion followed by in-solution propionylation of peptides

A 20 µg portion of histone extract dissolved in water was placed in a YM-10 Microcon filter unit (Millipore) with 300 µL of 50 mM TEAB (pH 8.5), centrifuged (14 000 g, 30 min, 25 °C) and washed three times with 200 µL of 50 mM TEAB. The sample was diluted with 50 mM TEAB to a volume of 30 µL then 2 µL of NH_4OH was added. A 10 µL portion of propionylation reagent, freshly prepared for each batch of three samples by mixing propionic anhydride and isopropanol in a 1:3 ratio, was immediately added to the sample. The pH was adjusted to 8–9 by NH_4OH, then the sample was incubated in a thermomixer (50 °C, 700 rpm, 40 min) and centrifuged (14 000 g, 10 min, 25 °C). The second round of propionylation was carried out with the same protocol. After derivatization, the sample was washed three times with 100 µL of 50 mM TEAB, and trypsin diluted in 50 µL of 50 mM TEAB was added in a 1:40 enzyme:protein ratio. Following overnight digestion at 37 °C, the digest was collected by centrifugation (14 000 g, 10 min, 25 °C), subjected to two additional washes with 50 µL of 50 mM TEAB, then concentrated in the SpeedVac to 5–10 µL and diluted with 50 mM TEAB to a volume of 20 µL. Peptides were propionylated at N-termini as follows. One µL of NH_4OH was added to the sample, then 5 µL of the propionylation reagent. The pH was adjusted to 8–9 by NH_4OH. The sample was incubated at 37 °C for 40 min, concentrated in the SpeedVac to 5 µL, reconstituted in 20 µL of 50 mM TEAB and propionylated at the peptide level again. The sample was then dried in the SpeedVac overnight and dissolved in 50 µL of 50% MeCN. The solution was concentrated in the SpeedVac to 20 µL and the peptide concentration was determined

using a Micro BCA™ Protein Assay Kit. Finally, before LC-MS/MS analysis, the sample was acidified by adding FA to a final acid concentration of 1%.

1.2 Mass spectrometric analysis of mammalian histone samples

1.2.1 LC-MS/MS analysis of derivatized histone peptides

The acquired peptide mixtures were analyzed by LC-MS/MS using a RSLCnano system (Thermo Fisher Scientific) connected on-line to an Impact II Ultra-High Resolution Qq-Time-Of-Flight mass spectrometer equipped with a CaptiveSpray nanoBooster ion source (Bruker). Portions of the solutions containing 40 ng of the derivatized human peptides (prepared as described above) were injected into the LC system, respectively. Prior to LC separation, peptides were concentrated online on a trapping column (100 µm × 30 mm), filled with 3.5-µm X-Bridge BEH 130 C18 sorbent (Waters), that had been equilibrated (together with the analytical column) with the initial mobile phase before injecting the sample into the sample loop. The peptides were separated using an Acclaim Pepmap100 C18 column; 3 µm particles, 75 µm × 500 mm; Thermo Fisher Scientific analytical column. The gradient elution was as follows: 0–90 min, 1–70% B; 90–100 min, 70–98% B; 100–120 min, 98% B (where the mobile phases A and B consisted of 0.1% FA in water and in 80% MeCN, respectively). After each injection, the flow rate was set at 500 nL/min for 13 minutes to load peptides on the column, then linearly decreased to 300 nL/min over 2 minutes. The analytical column's outlet was directly connected to the NanoBooster ion source, which was filled with MeCN. MS and MS/MS spectra were acquired in a data-dependent strategy with 3 s cycle time. The mass range was set to 150-2200 m/z and precursors were selected from 300 to 2000 m/z. The acquisition speed of MS and MS/MS scans (the latter varied according to precursor intensity) was 2 and 4-16 Hz, respectively. DataAnalysis software (version 4.2 SR1; Bruker) was used for pre-processing the mass spectrometric data (including recalibration, compound detection and charge deconvolution).

1.2.2 Database searches and quantification

In-house Mascot search engine (version 2.4.1; Matrixscience) was used to search for matches to exported MS/MS spectra obtained from analyses of human samples in the UniProtKB Human (version 2017_02; 21031 protein sequences), in-house Histone Human (version 2017_02; 114 protein sequences in total) and cRAP contaminants databases. Settings for all searches included semispecific Arg-C enzyme specificity and up to two missed cleavages. In addition, the following variable modifications were set for searches against the Histone human database: methyl (R, K), dimethyl (K), trimethyl (K), propionyl (K, N-term, S, T, Y), acetyl (K, protein N-term) and deamidation (N, Q). Propionyl (K, N-term, S, T, Y), acetyl (K, protein N-term) and deamidation (N, Q) were set as variable modifications in UniProtKB Human database searches. Mass tolerances of peptides and MS/MS fragments for MS/MS ion searches were 20 ppm and 0.05 Da, respectively. Manual peak labelling and peptide precursor area calculation were done via Skyline 3.6 software. A spectral library was created using the Proteome discoverer platform (version 1.4; Thermo Fisher Scientific). Only peptides with statistically significant peptide scores ($p < 0.01$) were included.

2 Supplementary Figures and Tables

2.1 Supplementary Figures

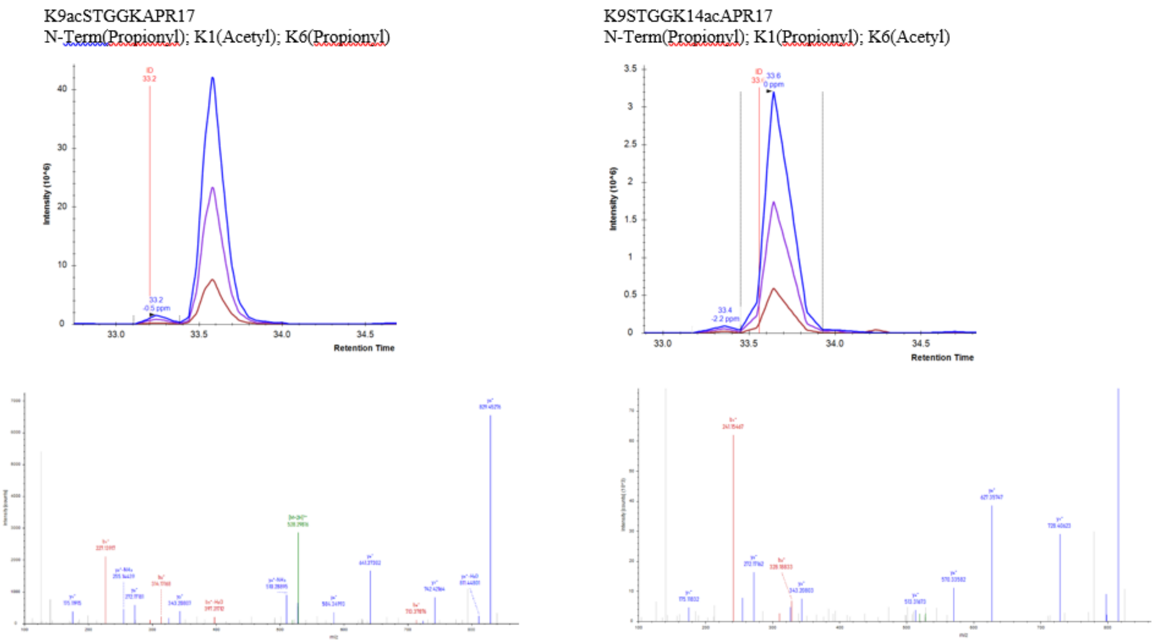

Fragment Matches

Value Type: Theo. Mass [Da]

Ion Series | Neutral Losses | Precursor Ions

#1	b+	b2+	Seq.	y+	y2+	#2
1	227.13903	114.07315	K-Propiony...			9
2	314.17106	157.58917	S	829.45269	415.22998	8
3	415.21874	208.11301	T	742.42066	371.71397	7
4	472.24021	236.62374	G	641.37298	321.19013	6
5	529.26168	265.13448	G	584.35151	292.67939	5
6	713.38286	357.19507	K-Propionyl	527.33004	264.16866	4
7	784.41998	392.71363	A	343.20885	172.10806	3
8	881.47275	441.24001	P	272.17173	136.58950	2
9			R	175.11896	88.06312	1

Fragment Matches

Value Type: Theo. Mass [Da]

Ion Series | Neutral Losses | Precursor Ions

#1	b+	b2+	Seq.	y+	y2+	#2
1	241.15468	121.08098	K-Propiony...			9
2	328.18671	164.59699	S	815.43704	408.22216	8
3	429.23439	215.12083	T	728.40501	364.70614	7
4	486.25586	243.63157	G	627.35733	314.18230	6
5	543.27733	272.14230	G	570.33586	285.67157	5
6	713.38286	357.19507	K-Acetyl	513.31439	257.16083	4
7	784.41998	392.71363	A	343.20885	172.10806	3
8	881.47275	441.24001	P	272.17173	136.58950	2
9			R	175.11896	88.06312	1

Supplementary Figure 1. A representative separation of positional isomers.

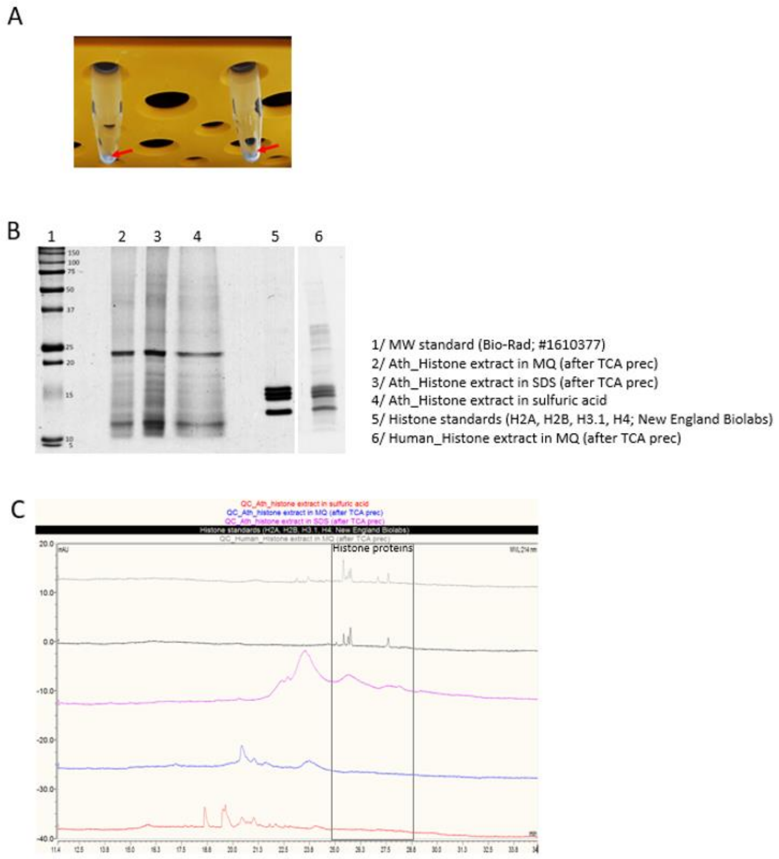

Supplementary Figure 2. Plant-specific features of histone preparation prior to LC-MS/MS. **(A)** Plant histone precipitate obtained from histone extract in sulfuric acid. Histone extract containing 20 μg of protein was precipitated using 25% trichloracetic acid (TCA), washed with 50 mM HCl in acetone, then with acetone, and dried. **(B)** SDS-PAGE of histone samples. Histone extract in sulfuric acid (SA) was divided into three identical aliquots, two of them were precipitated using TCA and re-dissolved either in MilliQ water (MQ) or 5 % SDS (SDS). Even though the same volume of the samples were loaded on the gel, line 4 was distorted due to the nature of SA sample. To demonstrate the higher degree of co-extraction and co-precipitation of non-histone proteins in case of plant samples, human histone extract after TCA precipitation re-dissolved in MilliQ water is presented. Human recombinant histone standards are also presented. **(C)** HPLC of histone samples. A $\frac{1}{14}$ portion of each histone sample was used for quality control – SA (red), MQ (blue), or SDS (pink). Considerable amount of protein or non-protein contaminants was observed in both SA and MQ chromatograms while histone protein peaks were not detected. Due to presence of SDS, poor quality of SDS sample separation was observed. The histone proteins are likely to be hidden in the broad peaks. Human recombinant histone standards and human histone extract after TCA precipitation re-dissolved in MilliQ water are also presented (black and grey, respectively).

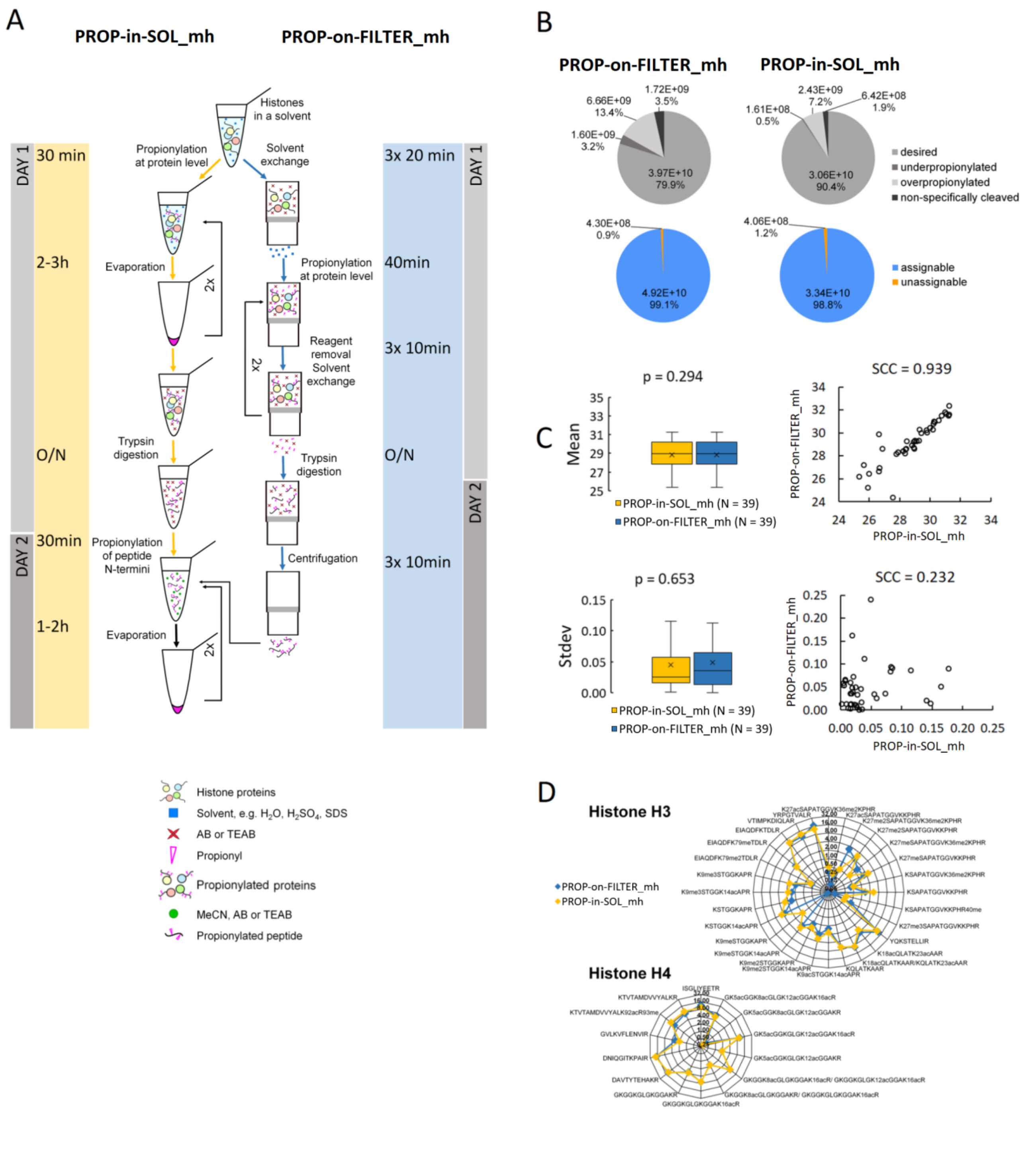

Supplementary Figure 3. Chemical derivatization technique for histone proteins adapted to on-membrane performance using mammalian histone extracts. **(A)** An illustrative scheme of the PROP-on-FILTER_mh workflow including comparison with a commonly used in-solution derivatization (PROP-in-SOL_mh). **(B)** PROP-on-FILTER_mh performance compared to PROP-in-SOL_mh approach. Grayscale pie charts showing proportions of identified histone H3 and H4 peptides in four categories – desired (peptides cleaved and propionylated as expected), underpropionylated (peptides

with at least one unmodified amino group on lysine residue or N-terminus), overpropionylated (peptides with at least one propionylated hydroxyl group on S, T or Y residue), non-specifically cleaved (peptides with cleavage at lysine C terminus or missed cleavage at arginine C terminus). Color pie charts showing proportions of assignable peptides, i.e. peptides enabling correct quantification. **(C)** Box-plots and scatter-plots of the means and standard deviations of abundances of histone H3 and H4 peptide forms detected in the samples. The boxplots show extremes, interquartile ranges and medians (N=39). Means and standard deviations were compared by Mann-Whitney tests (p-values) and Spearman's correlation coefficients (SCC values). **(D)** Radar charts showing relative abundances of individual peptide forms of histone H3 and H4 (means; N = 2), determined from the ratio of the XIC peak areas of particular assignable products to the summed XIC peak areas of the total pool of all quantified H3 or H4 peptides, respectively. The Y axes have a binary logarithm scale, with zero located in the center.

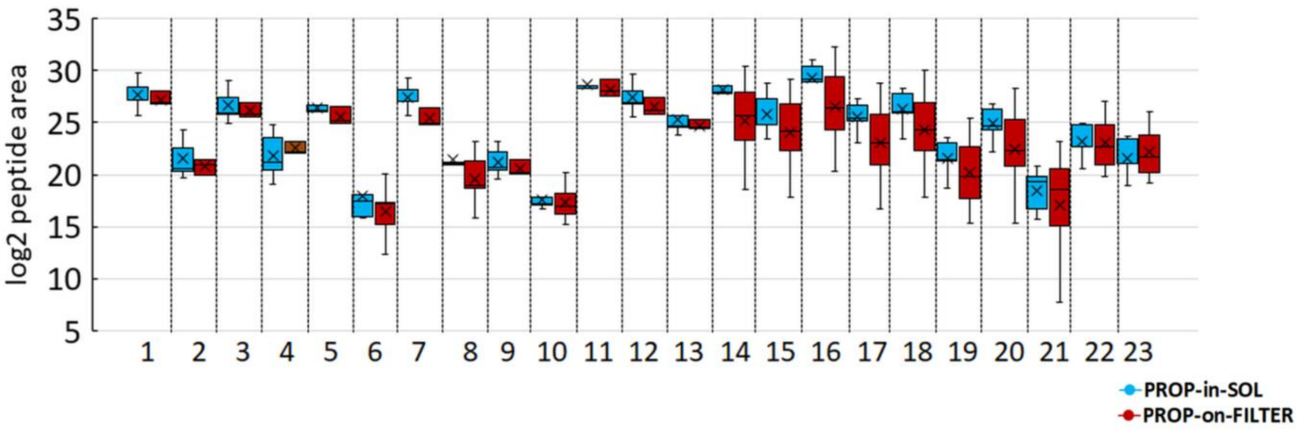

Supplementary Figure 4. Comparison of PROP-in-SOL and PROP-on-FILTER performance in term of inter-sample variability of selected plant histone mark levels. The distribution of log2-transformed peptide precursor XIC peak areas is represented by a pair of boxplots. The boxplots show extremes, interquartile ranges and medians obtained from analyses of five samples. The numbers on the x-axis correspond to the peptide order in the Supplementary Table 1.

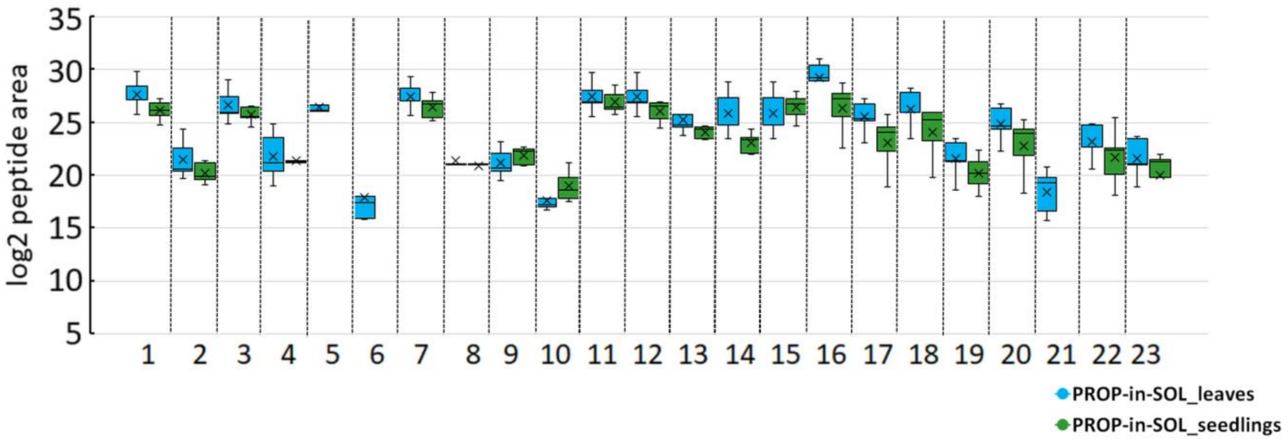

Supplementary Figure 5. PROP-in-SOL performance in term of inter-sample variability of selected plant histone mark levels in seven weeks old leaves and seven days old seedlings. The distribution of log2-transformed peptide precursor XIC peak areas is represented by a pair of boxplots. The boxplots show extremes, interquartile ranges and medians obtained from analyses of five samples. The numbers on the x-axis correspond to the peptide order in the Supplementary Table 1 and 2, respectively.

2.2 Supplementary Tables

Supplementary Table 1 Comparison of PROP-on-FILTER and PROP-in-SOL performance using plant histone extracts. Log2-transformed peptide precursor areas and relative abundances (RA) of individual peptide forms of histone H3 and H4 are presented for each replicate. RA was determined as the ratio of the XIC peak areas of particular assignable products to the summed XIC peak areas of the total pool of all quantified H3 or H4 peptides, respectively.

			PROP-in-SOL												PROP-on-FILTER											
Histone	Peptide modified form	#	log2-transformed peptide precursor area						RA (%)						log2-transformed peptide precursor area						RA (%)					
			repl. 1	repl. 2	repl. 3	repl. 4	repl. 5	median	repl. 1	repl. 2	repl. 3	repl. 4	repl. 5	median	repl. 1	repl. 2	repl. 3	repl. 4	repl. 5	median	repl. 1	repl. 2	repl. 3	repl. 4	repl. 5	median
H3	K9STGGKAPR17	1	27.13	27.17	25.71	28.43	29.84	27.66	11.96	12.13	11.62	16.64	12.38	12.13	26.75	23.88	30.51	28.02	26.88	27.21	21.40	22.55	14.30	15.21	18.02	18.02
	K9acSTGGKAPR17	2	20.36	20.58	19.68	22.55	24.34	21.50	0.11	0.13	0.18	0.28	0.27	0.18	19.98	17.12	24.40	21.47	20.97	20.79	0.20	0.21	0.21	0.16	0.30	0.21
	K9STGGK14acAPR17	3	25.81	25.93	24.88	27.39	29.03	26.61	4.80	5.13	6.52	8.09	7.09	6.52	25.84	22.98	29.41	26.92	25.58	26.15	11.33	12.04	6.70	7.09	7.30	7.30
	K9acSTGGK14acAPR17	4	19.05	20.43	21.19	23.55	24.83	21.81	0.04	0.11	0.51	0.56	0.39	0.39	22.10	19.70	25.76	23.22	22.13	22.58	0.85	1.24	0.53	0.54	0.67	0.67
	K9me1STGGKAPR17	5	26.03	26.11	24.55	26.64	28.71	26.41	5.60	5.80	5.19	4.78	5.66	5.60	24.93	21.94	29.03	26.50	25.16	25.51	6.06	5.86	5.12	5.29	5.44	5.44
	K9me1STGGK14acAPR17	6	17.43	18.05	15.95	15.79	22.62	17.97	0.01	0.02	0.01	0.00	0.08	0.01	17.14	15.15	20.08	17.36	12.37	16.42	0.03	0.05	0.01	0.01	0.00	0.01
	K9me2STGGKAPR17	7	27.06	27.09	25.68	28.19	29.34	27.47	11.39	11.42	11.41	14.01	8.80	11.41	24.77	21.48	29.23	26.39	24.97	25.37	5.43	4.27	5.89	4.90	4.79	4.90
	K9me2STGGK14acAPR17	8	21.11	21.13	20.37	20.97	23.50	21.42	0.18	0.18	0.29	0.09	0.15	0.18	18.96	15.81	23.22	21.26	18.64	19.58	0.10	0.08	0.09	0.14	0.06	0.09
	K9me3STGGKAPR17	9	20.41	20.65	19.53	22.12	23.13	21.17	0.11	0.13	0.16	0.21	0.12	0.13	20.12	16.87	24.03	21.39	20.22	20.53	0.22	0.18	0.16	0.15	0.18	0.18
	K9me3STGGK14acAPR17	10	17.86	17.04	17.19	16.73	19.13	17.59	0.02	0.01	0.03	0.00	0.01	0.01	16.26	15.21	20.15	18.16	16.96	17.35	0.01	0.06	0.01	0.02	0.02	0.02
	K18QLATKAAR26	11	28.55	28.52	27.02	28.29	30.96	28.67	32.09	30.77	28.88	15.05	26.91	28.88	27.52	24.61	31.74	29.18	27.98	28.20	36.30	37.26	33.69	33.82	38.47	36.30
	K18acQLATKAAR26/ K18QLATK23acAAR26	12	26.85	26.90	25.57	28.07	29.69	27.41	9.87	10.00	10.54	12.90	11.16	10.54	25.74	22.84	30.22	27.46	26.18	26.49	10.61	10.94	11.73	10.27	11.08	10.94
	K18acQLATK23acAAR26	13	24.53	24.71	23.76	25.72	27.62	25.27	1.98	2.20	3.00	2.54	2.67	2.54	24.49	21.52	27.92	25.26	24.42	24.72	4.47	4.37	2.39	2.25	3.26	3.26
	K27me1SAPATGGVKKPHR40	14	27.83	27.87	26.44	28.49	30.40	28.20	19.49	19.67	19.26	17.25	18.25	19.26	23.35	18.56	30.41	27.89	25.63	25.17	2.02	0.56	13.34	13.85	7.55	7.55
	K27me3SAPATGGVKKPHR40	15	24.77	24.78	23.43	27.30	28.81	25.82	2.34	2.30	2.40	7.57	6.06	2.40	22.31	17.79	29.21	26.75	24.23	24.06	0.99	0.33	5.83	6.29	2.85	2.85
H4	G4KGGKGLGKGGAKR17	16	29.18	28.97	26.60	31.04	30.40	29.24	80.30	79.34	78.63	77.05	74.67	78.63	24.27	20.32	32.28	29.35	26.42	26.53	65.36	75.65	71.25	72.29	69.62	71.25
	G4KGGK8acGLGKGGAKR17/ G4KGGKGLGK12acGGAKR17	17	25.38	25.19	23.07	27.24	26.72	25.52	5.80	5.80	6.82	5.56	5.83	5.80	20.93	16.67	28.74	25.78	23.05	23.04	6.48	6.04	6.11	6.06	6.74	6.11
	G4KGGKGLGKGGAK16acR17	18	25.99	25.97	23.43	28.28	27.80	26.29	8.81	9.89	8.71	11.38	12.28	9.89	22.31	17.86	30.01	26.96	24.25	24.28	16.80	13.72	14.74	13.73	15.49	14.74
	G4K5acGGKGLGK12acGGAKR17/ G4KGGK8acGLGK12acGGAKR17	19	21.40	21.25	18.64	23.51	23.06	21.57	0.37	0.38	0.32	0.42	0.46	0.38	17.64	15.28	25.37	22.74	19.78	20.16	0.66	2.30	0.59	0.74	0.70	0.70
	G4KGGK8acGLGKGGAK16acR17	20	24.66	24.33	22.24	26.78	26.35	24.87	3.50	3.18	3.83	4.04	4.51	3.83	20.76	15.28	28.26	25.25	22.31	22.37	5.74	2.30	4.38	4.22	4.03	4.22
	G4K5acGGK8acGLGK12acGGAKR17	21	15.70	19.27	16.64	20.81	19.81	18.45	0.01	0.10	0.08	0.06	0.05	0.06	7.78	N/F	23.14	19.71	17.43	17.01	N/F	0.00	0.13	0.09	0.14	0.11
	G4K5acGGKGLGK12acGGAK16acR17	22	22.72	22.66	20.61	24.89	24.78	23.13	0.92	1.00	1.24	1.09	1.52	1.09	19.82	N/F	26.99	24.11	21.35	23.07	3.00	0.00	1.82	1.91	2.07	1.91
	G4K5acGGK8acGLGK12acGGAK16acR17	23	21.09	21.01	18.88	23.46	23.63	21.61	0.30	0.32	0.37	0.40	0.68	0.37	19.20	N/F	26.03	23.02	20.44	22.17	1.95	0.00	0.94	0.90	1.10	0.94

Supplementary Table 2 PROP-in-SOL performance using plant histones extracted from seven days old seedlings. Log2-transformed peptide precursor areas and relative abundances (RA) of individual peptide forms of histone H3 and H4 are presented for each replicate. RA was determined as the ratio of the XIC peak areas of particular assignable products to the summed XIC peak areas of the total pool of all quantified H3 or H4 peptides, respectively.

			PROP-in-SOL_SEEDLINGS											
**Histone**	**Peptide modified form**	**#**	**log2-transformed peptide precursor area**						**RA (%)**					
			**repl. 1**	**repl. 2**	**repl. 3**	**repl. 4**	**repl. 5**	**median**	**repl. 1**	**repl. 2**	**repl. 3**	**repl. 4**	**repl. 5**	**median**
**H3**	K9STGGKAPR17	1	27.20	24.76	26.18	26.85	25.65	26.18	11.10	10.29	10.47	15.05	17.03	11.10
	K9acSTGGKAPR17	2	21.17	19.16	19.88	21.34	19.60	19.88	0.17	0.21	0.13	0.33	0.26	0.21
	K9STGGK14acAPR17	3	26.59	24.58	25.51	26.44	25.43	25.51	7.30	9.10	6.62	11.34	14.56	9.10
	K9acSTGGK14acAPR17	4	21.25	21.23	20.62	22.39	21.43	21.25	0.18	0.89	0.22	0.68	0.91	0.68
	K9meSTGGKAPR17	5	N/Q	N/Q	N/Q	N/Q	N/Q	N/Q	N/Q	N/Q	N/Q	N/Q	N/Q	N/Q
	K9meSTGGK14acAPR17	6	N/Q	N/Q	N/Q	N/Q	N/Q	N/Q	N/Q	N/Q	N/Q	N/Q	N/Q	N/Q
	K9me2STGGKAPR17	7	27.81	25.50	26.70	27.00	25.11	26.70	16.97	17.12	15.04	16.70	11.67	16.70
	K9me2STGGK14acAPR17	8	22.13	21.05	20.95	21.01	19.56	21.01	0.33	0.79	0.28	0.26	0.25	0.28
	K9me3STGGKAPR17	9	22.66	20.99	22.25	22.43	20.89	22.25	0.48	0.75	0.69	0.71	0.63	0.69
	K9me3STGGK14acAPR17	10	21.16	19.33	17.92	17.49	N/Q	18.62	0.17	0.24	0.03	0.02	0.00	0.03
	K18QLATKAAR26	11	28.55	26.21	27.60	26.45	25.77	26.45	28.41	27.98	28.11	11.45	18.44	27.98
	K18acQLATKAAR26/K18QLATK23acAAR26	12	26.95	25.36	26.82	26.57	24.45	26.57	9.38	15.53	16.40	12.38	7.42	12.38
	K18acQLATK23acAAR26	13	24.69	23.36	24.36	24.58	23.51	24.36	1.96	3.90	2.97	3.13	3.85	3.13
	K27meSAPATGGVKKPHR40	14	24.36	22.07	23.42	23.61	21.95	23.42	17.89	9.60	16.04	20.21	17.83	17.83
	K27me3SAPATGGVKKPHR40	15	27.88	24.66	26.79	27.27	25.72	26.79	4.10	2.02	1.43	6.13	5.85	4.10
**H4**	G4KGGKGLGKGGAKR17	16	28.76	25.53	27.73	27.20	22.56	27.20	67.97	70.50	64.60	65.46	74.83	67.97
	G4KGGK8acGLGKGGAKR17/G4KGGKGLGK12acGGAKR17	17	25.72	22.26	24.55	24.05	18.92	24.05	8.25	7.32	7.11	7.38	6.00	7.32
	G4KGGKGLGKGGAK16acR17	18	25.95	23.26	25.96	25.23	19.80	25.23	9.70	14.65	18.96	16.73	11.04	14.65
	G4K5acGGKGLGK12acGGAKR17/G4KGGK8acGLGK12acGGAKR17	19	22.42	18.00	20.85	19.61	N/Q	20.23	0.84	0.38	0.55	0.34	0.00	0.38
	G4KGGK8acGLGKGGAK16acR17	20	25.25	21.86	24.34	23.92	18.30	23.92	5.97	5.55	6.18	6.76	3.91	5.97
	G4K5acGGK8acGLGK12acGGAKR17	21	N/Q	N/Q	N/Q	N/Q	N/Q	N/Q	N/Q	N/Q	N/Q	N/Q	N/Q	N/Q
	G4K5acGGKGLGK12acGGAK16acR17	22	25.42	20.07	22.59	22.40	18.15	22.40	6.68	1.61	1.83	2.35	3.53	2.35
	G4K5acGGK8acGLGK12acGGAK16acR17	23	21.94	N/Q	21.36	21.13	15.78	21.24	0.60	0.00	0.78	0.97	0.68	0.68

Supplementary Table 3 Summary of PROP-in-SOL and PROP-on-FILTER features.

Derivatization method	Matrix	Contaminant removal: Derivatization agent rest	Contaminant removal: Buffer exchange	Protein digestion	Derivatization efficiency	Data variability: Histone H3	Data variability: Histone H4	Developmental stages tested	Time needed per derivatization round (hours)
Prop-in-SOL	Vial	Evaporation Ultrafiltration	Ultrafiltration	Filter unit	> 90 %	Low	Low	7d seedlings 7w leaves	~ 4
Prop-on-FILTER	Filter unit	Ultrafiltration	Ultrafiltration	Filter unit	> 90 %	Medium	High	7w leaves	~ 3
